# Rac3 induces a molecular pathway triggering breast cancer cell aggressiveness: differences in MDA-MB-231 and MCF-7 breast cancer cell lines

**DOI:** 10.1186/1471-2407-13-63

**Published:** 2013-02-06

**Authors:** Caroline Gest, Ulrich Joimel, Limin Huang, Linda-Louise Pritchard, Alexandre Petit, Charlène Dulong, Catherine Buquet, Chao-Quan Hu, Pezhman Mirshahi, Marc Laurent, Françoise Fauvel-Lafève, Lionel Cazin, Jean-Pierre Vannier, He Lu, Jeannette Soria, Hong Li, Rémi Varin, Claudine Soria

**Affiliations:** 1 Laboratoire MERCI - EA3829, Faculté de Médecine et de Pharmacie, Université de Rouen, Rouen, France; 2 INSERM UMR-S 728, IUH, Hôpital Saint-Louis, Paris, France; 3 Université Paris-Sud and CNRS FRE 3377, CEA-Saclay, Gif-sur-Yvette, France; 4 Department of Onco-Haematology, UMRS 872, CNRS, E 18, INSERM, Université Paris VI, Paris, France; 5 INSERM U553, Hôpital Saint-Louis, Paris, France; 6 Unité Fonctionnelle Oncologie Médicale, Hôtel-Dieu, Assistance Publique-Hôpitaux de Paris (AP-HP), Paris, France; 7 Present address: Unité Fonctionnelle Oncologie Médicale, Hôtel-Dieu, Assistance Publique-Hôpitaux de Paris (AP-HP), Paris, France

**Keywords:** Breast cancer, Cancer aggressiveness, Rac3 GTPases, ERK, NF-κB

## Abstract

**Background:**

Rho GTPases are involved in cellular functions relevant to cancer. The roles of RhoA and Rac1 have already been established. However, the role of Rac3 in cancer aggressiveness is less well understood.

**Methods:**

This work was conducted to analyze the implication of Rac3 in the aggressiveness of two breast cancer cell lines, MDA-MB-231 and MCF-7: both express Rac3, but MDA-MB-231 expresses more activated RhoA. The effect of Rac3 in cancer cells was also compared with its effect on the non-tumorigenic mammary epithelial cells MCF-10A. We analyzed the consequences of Rac3 depletion by anti-Rac3 siRNA.

**Results:**

Firstly, we analyzed the effects of Rac3 depletion on the breast cancer cells’ aggressiveness. In the invasive MDA-MB-231 cells, Rac3 inhibition caused a marked reduction of both invasion (40%) and cell adhesion to collagen (84%), accompanied by an increase in TNF-induced apoptosis (72%). This indicates that Rac3 is involved in the cancer cells’ aggressiveness. Secondly, we investigated the effects of Rac3 inhibition on the expression and activation of related signaling molecules, including NF-κB and ERK. Cytokine secretion profiles were also analyzed. In the non-invasive MCF-7 line; Rac3 did not influence any of the parameters of aggressiveness.

**Conclusions:**

This discrepancy between the effects of Rac3 knockdown in the two cell lines could be explained as follows: in the MDA-MB-231 line, the Rac3-dependent aggressiveness of the cancer cells is due to the Rac3/ERK-2/NF-κB signaling pathway, which is responsible for MMP-9, interleukin-6, -8 and GRO secretion, as well as the resistance to TNF-induced apoptosis, whereas in the MCF-7 line, this pathway is not functional because of the low expression of NF-κB subunits in these cells. Rac3 may be a potent target for inhibiting aggressive breast cancer.

## Background

The proliferative and invasive abilities of breast cancer cells are correlated with aggressiveness and poor prognosis. Therefore, understanding the molecular mechanisms involved in the aggressiveness is important for the identification of new therapeutic targets. It was previously shown that Rho and Rac GTPases promote cancer progression [1]. Indeed, increased RhoA expression was described in various human tumours to correlate with poor prognosis [2,3]. Rac1 is over-expressed in various tumours, accumulating evidence indicates that Rac1-dependent cell signaling is important for malignant transformation [4], and overexpression of Rac1 correlates with breast cancer progression. The role of Rho family proteins in cancer cell aggressiveness involves both cytoskeleton organization, which control several processes relevant to cell migration including adhesion of cells to the extracellular matrix, and activation of cell signaling processes leading to the activation of transcription factors. The precise relationships between the various Rho GTPases and their effects on cell locomotion are still unclear. Nobes and Hall [5] showed that the small GTPases Rho, Rac and Cdc42 coordinate the spatial and temporal changes in the actin cytoskeleton that lead to cellular movement. They proposed that the activation of Cdc42 leads to Rac activation, and that Rac subsequently activates Rho. However, Rottner *et al.*[6] suggested that Rac and Rho influence the development of focal contacts and focal complexes, respectively, through mutually antagonistic pathways. Finally, Sanders *et al.*[7] proposed a unidirectional signaling cascade, from Rac towards Rho, since only activated Rac results in abrogation of Rho activity. They also indicated that Rho activity occurs independently of Rac-induced cytoskeletal changes and cell spreading.

The subgroup of Rac GTPases contains 3 major proteins: Rac1 is ubiquitously expressed, Rac2 is specific for haematopoietic cells, and Rac3 is enriched in the brain but is also expressed in a wide range of tissues [8].

Despite the high homology in amino-acid sequence (92%) between Rac1 and Rac3, Rac3 differs from Rac1 in the COOH terminal region, which is involved in Rac localization and regulatory protein binding [8,9]. However, most of the literature addressing the role of Rac in cancer aggressivity concerns Rac1, and studies on the role of Rac3 in cancer progression are far less abundant. That said, Baugher *et al.*[10] have reported that both Rac1 and Rac3 activation are involved in the invasive and metastatic phenotype of human breast cancer cells. To demonstrate this, the authors used dominant active and negative mutants of Rac1 and Rac3. It is known that dominant negative Rac mutants are highly promiscuous in binding and sequestering various guanine nucleotide exchange factors, or GEFs [11]. It is thus difficult to address, by this method, the precise functions of these highly homologous proteins.

The aim of our study was two fold. Firstly, we sought to clarify the role of Rac3 in breast cancer cell aggressiveness. Rac3 is expressed in many types of cells, and although its physiological activity seems to be dispensable in normal tissues [12], increases in its activation nevertheless lead to lesions in mammary tissue [13]. Moreover, Rac3 proteins are frequently overexpressed and mutated in human brain tumours, which may be associated with aggressive tumour behaviour [14]; and transfection of a dominant active variant of Rac3 into low metastatic breast cancer cells leads to an increase of cell invasiveness [10]. Secondly, we wished to examine the biological mechanisms by which expression of Rac3 may exert an effect in cells in which RhoA is overexpressed and constitutively activated or not. To answer these questions, we examined the consequences of Rac3 inhibition by siRNA in two breast cancer cell lines: MDA-MB-231 (Estrogen Receptor (ER) negative), an aggressive line that overexpresses constitutively activated RhoA; and MCF-7 (ER positive), which is not invasive. The highly specific siRNA approach used here represents a more reliable tool than those previously used for loss-of-function studies. We analyzed the effects of this inhibition on cell proliferation, invasiveness, adhesion to extracellular matrix, vasculogenic mimicry, and apoptosis inhibition, as criteria of cancer cell aggressivity.

## Methods

### Cells

MDA-MB-231 cells were grown in RPMI 1640 medium with 10% fetal bovine serum (FBS) (Eurobio), 2 mM L-glutamine. MCF-7 cells were grown in H-DMEM medium with 10% FBS, 4 mM L-glutamine. Both cell lines were obtained from ECACC. The non-tumorigenic mammary epithelial cell line MCF-10A was purchased from ATCC and cultured with HAM's F12/DMEM (1:1) medium supplemented with 20 ng/ml EGF, 5% horse serum, 100 ng/ml cholera toxin, 0.5 μg/ml, hydrocortisone and 10 mg/ml insulin.

All cultures contained 100 IU/ml penicillin and 100 μg/ml streptomycin (Eurobio) and were incubated at 37°C in a humidified 5% CO_2_ atmosphere.

### siRNA transfection

Specific siRNA directed against human Rac3 was designed using the criteria established by Tuschl [15]. Candidate sequences were compared with cDNA sequences and their specificity verified in the non-redundant human DNA database using a BLAST algorithm [accession through NCBI]. The Rac3 siRNA selected was: sense 5’-CUGACGUCUUUCUGAUCUG-3’, antisense 5’-CAGAUCAGAAAGACGUCAG-3’. Eurogentech negative control siRNA was used as control. siRNAs (10 nM) were introduced into cells by INTERFERin™-mediated transfection (Ozyme).

### Reverse-Transcriptase–Polymerase Chain Reaction (RT-PCR) for Rac3

Total mRNA was extracted with a SV Total RNA Isolation System (Promega), and specific transcripts amplified using these primers: Rac3 (forward, 5’-ACGGGAAACCAGTCAACT-3’; reverse 5’-GCAGCCGCTCAATGGT-3’) [16]; GAPDH (forward, 5’-AAGGTCATCCCTGAGCTGAA -3’; reverse, 5’-CCCCTCTTCAAGGGGTCTAC -3’). RT-PCR was carried out in a GeneAmp PCR system 9700 (Applied Biosystems).

### Western Blot

For protein extractions, 2 × 10^6^ cells were seeded into 75 cm^2^ flasks. 48 h and/or 72 h after transfection, proteins were extracted using RIPA buffer containing protease and phosphatase inhibitor cocktail. The protein concentration was determined using a BCA Protein Assay kit (Pierce). Protein fractions were separated by SDS-PAGE, then transferred onto polyvinylidene difluoride membranes (Amersham) using a dry transfer system (Invitrogen). Membranes were blocked with skim milk, probed using anti-Rac3 (ProteinTech Group), anti-Rac1 (Upstate), anti-RhoA (Santa Cruz), anti-Cdc42 (Cell Signaling Technology), anti-ERK (Cell Signaling Technology), anti-p105/p50 (Santa Cruz), anti-p65 (Abcam), anti-IKKα (Cell Signaling Technology), anti-IKKβ (Cell Signaling Technology), anti-IκBα (Cell Signaling Technology), anti-Histone H3 (Cell Signaling Technology) and anti-GAPDH (Sigma Aldrich). Membranes blocked with BSA were also probed using anti-phospho ERK (Cell Signaling Technology), anti-phospho IKKα/β and anti-phospho IκBα (Cell Signaling Technology) primary antibodies. The detection was done using a secondary peroxidase-conjugated antibody (Dako). After washing, bound antibody was detected with Immobilon western chemiluminescente HRP substrate (Millipore). Chemiluminescent emissions were captured on X-ray films (Amersham).

For NF-κB detection, 10^6^ MDA-MB-231 cells were seeded into 75 cm^2^ flasks. 72 h after transfection, nuclear extracts and cytoplasmic fractions were separated using an NE-PER kit (Pierce).

### Detection of RhoA and Cdc42 activation

Cells were seeded (2 × 10^6^) into 75 cm^2^ flasks in medium with 5% FBS. 72 h after transfection, the cells were lysed, and activated RhoA or Cdc42 was measured with a G-Lisa kit, following the manufacturer’s instructions (Cytoskeleton). Briefly, the active GTP-bound GTPase in the biological sample binds to the effector-coated plates, but the inactive, GDP-bound form is removed during washing. Bound active RhoA or Cdc42 is then detected by incubation with a specific antibody conjugated to peroxidase. Results are expressed as percentage of control, mean ± S.E.

### Immunofluorescence

Cells were seeded (2 × 10^4^/well) in 8-well Lab-Tek slides (Nunc, Thermo Fisher Scientific). After 48 h of siRNA treatment, cells were fixed with 4% paraformaldehyde and permeabilised with 0.2% Triton; actin filaments were visualized by tetramethylrhodamine isothiocyanate (TRITC) labelled phalloidin (Sigma Aldrich) and examined under a Leica model DM 5500B microscope.

### Wound healing assay (scratch test)

Cells were seeded in triplicate into 24-well plates at 10^5^ cells/well in medium containing 2% FBS, and transfected with siRNA anti-Rac3 24 h later. After a further 48 h of incubation in presence of the siRNA, when the cells in all treatment groups had reached confluence, the monolayer was subjected to a scratch test to assess directional motility in healing the “wound”. Wound closure was observed by microcinematography (Zeiss) during the following 48 h, with photographs taken every 20 min at 50 X magnification. Axiovision software was used to quantify the linear advancement of the migration front in closing the scratch; results are presented as % wound closure, calculated from the mean distances between the two migration fronts at 0, 12, 24 and 48 h after wounding.

### Adhesion on collagen type I under flow conditions

10^6^ cells were seeded per 75 cm^2^ flask, treated with anti-Rac3 or control siRNA for 48 h, detached with Versene (Invitrogen), counted, centrifuged, and resuspended at a final concentration of 10^7^ cells/ml before labelling with CellTracker™ Red CMTPX (Invitrogen) for 20 min in adhesion buffer (20 mM HEPES, 150 mM NaCl, 5 mM KCl, 1 mM MgSO_4_, and 1 mM MnCl_2_, pH 7.4). Blood samples collected on 0.13 M citrate (9 vol blood for 1 vol citrate) were centrifuged for 20 min at 800 rpm. Platelet-rich plasma (PRP) was collected and labelled with Calcein-AM (Interchim). PRP and cells were co-incubated for 20 min at 37°C in a 5% CO_2_ atmosphere [17].

Glass slides coated with type I collagen as described previously [18] were placed into a parallel plate perfusion chamber [19]. Blood containing Calcein-AM-labelled platelets (green) and CellTracker™-Red-labelled MDA-MB-231 cells (red) was perfused through the chamber for 10 min at a constant flow rate of 3 ml/min, corresponding to a shear rate of 1500 s^-1^, according to the chamber characteristics (5 mm wide and 0.2 mm high).

At the end of perfusion, the chamber was washed with PBS for 1 min at the same shear rate. The microscope was coupled to a 1394 (Scion Corporation) numeric high resolution camera directly linked to a computer equipped with the Scion Visiocapture acquisition software, and 20 random fields were recorded over a 1 cm^2^ area in the middle of the perfusion chamber. MDA-MB-231 tumour cell adhesion was estimated by measuring the area they covered using image J software.

### Invasion assay

Cells were cultivated in serum-free medium; after 48 h of siRNA treatment, treatment medium was aspirated and set aside, cells were washed once in PBS, detached with trypsin/EDTA solution, and resuspended in their respective treatment medium; then 1 × 10^6^ cells were seeded in the matrigel-coated insert of a Boyden chamber (BD Biosciences). The lower chamber was filled with 750 μl of medium containing 10% FBS to induce chemotaxis. 24 h later, the non-migrated cells in the upper chamber were gently scraped away, and adherent cells present on the lower surface of the insert were fixed with methanol, stained with 1% toluidine/1% borax solution, and counted in twenty randomly chosen fields from each chamber using Mercator software (Explora Nova).

### Capillary-like structure formation by breast cancer cells: vasculogenic mimicry (VM)

Cells were cultivated in culture medium complemented with 10% FBS; after 48 h of siRNA treatment, treatment medium was aspirated and set aside, cells were washed once in PBS, detached with trypsin/EDTA solution, and resuspended in their respective treatment medium; then 2 × 10^4^ of these breast cancer cells were seeded per well on growth-factor-rich Matrigel in 96-well plates to analyze the formation of capillary-like structures. After 4 h incubation, the wells were photographed at 100X magnification using an inverted light microscope. The extent of capillary-like tube formation was quantified by measuring the cumulative tube lengths and total number of intersections in three randomly chosen fields from each well using Image-Pro Plus software (Microvision Instruments).

### Cell proliferation/viability

Cells were seeded (6 × 10^3^/well) in 96-well plates in growth medium complemented with 5% FBS. Cell proliferation/viability was evaluated using a [3-(4,5-dimethylthiazol-2-yl)-5-(3-carboxymethoxyphenyl)-2-(4-sulfophenyl)-2H-tetrazolium, inner salt] (MTS, Promega) assay at 24, 48, 72 and 96 h after treatment. Cells were incubated with MTS in culture medium at 37°C for 2 h. Optical density was read at 490 nm using a PowerWave_x_ spectrophotometer (Bio-tek instruments, INC).

### TNFα-induced apoptosis

After 48 h of siRNA treatment, treatment medium was aspirated and set aside; cells were washed once in PBS, detached with trypsin/EDTA solution, resuspended in the treatment medium, and seeded at 2 × 10^4^ per well in 96-well plates; 3 h after seeding, cells were treated with 50 ng/ml of TNFα. 24 h later, apoptosis-induced DNA fragmentation was quantified using an ELISAPLUS cell death detection kit (Roche Diagnostics) by measuring the formation of histone-complexed DNA fragments (nucleosomes) present in the cytoplasm. Results are expressed as the adjusted absorbance, A_405_ minus A_490_.

### Cell cycle

To study the proportion of cells in different cell cycle phases, propidium iodide was used and detected by flow cytometry. Briefly, cells were treated with control or Rac3 siRNA. After 96 h of treatment, cells in the supernatant and adherent cells were collected, washed and fixed with cold ethanol. Cells were stored at −20°C until staining was performed. Cells were incubated with 500 μl of PBS containing 50 μg of RNase A (Sigma Aldrich) and 25 μg of propidium iodide (Sigma Aldrich) for 20 min in the dark. Cells were analysed with a BD FACSCalibur (BD Biosciences), and data were analysed using FlowJo® Mac 9.5.2. software.

### MMP secretion

MMP-9 was detected by zymography. Cells were treated for 48 h with siRNA in serum-free medium. Supernatants were collected by centrifugation, then kept frozen until analysis. For MMP assessment, supernatants were separated by electrophoresis on 7.5% polyacrylamide gels containing 10% SDS and gelatin (1 mg/ml) under nonreducing conditions. SDS was then removed from the gels by washing for 1 h in 2.5% Triton X-100 at room temperature. Gelatinase activity was developed overnight at 37°C in a buffer containing 50 mM Tris–HCl and 5 mM calcium chloride. Upon staining with 0.25% Coomassie blue R250 (Sigma), gelatinase activity was observed as clear bands against the blue background of the stained gelatin.

### Detection of cytokine secretion

The Human Cytokine Antibody Array III kit (RayBiotech) was used to evaluate 42 different cytokines. Briefly, 1 ml of undiluted supernatants harvested after 48 h of siRNA treatment of cells cultured in serum-free medium were incubated with arrayed antibody membranes, which were then exposed to the specific biotin-antibody cocktail, following the manufacturer's instructions. Signals were detected using labelled streptavidin by exposure on X-ray films. The relative amount of each cytokine present in the culture medium is presented as the percent increase or decrease of the spot intensity in the siRNA Rac3 medium compared to the control. The area density of the spots was evaluated using imageJ. Signals were normalized against the positive controls across membranes.

### Quantification

RT-PCR, western blot, zymography and cytokine array analysis was performed by ImageJ software (written in Java).

### Statistics

The Dunnett test was used for quantitative comparisons between treatments. All experiments were reproduced at least 3 times on different days unless specified otherwise.

## Results

### Anti-Rac3 siRNA transfection efficacy and action on Rho family proteins

**Figure 1:**
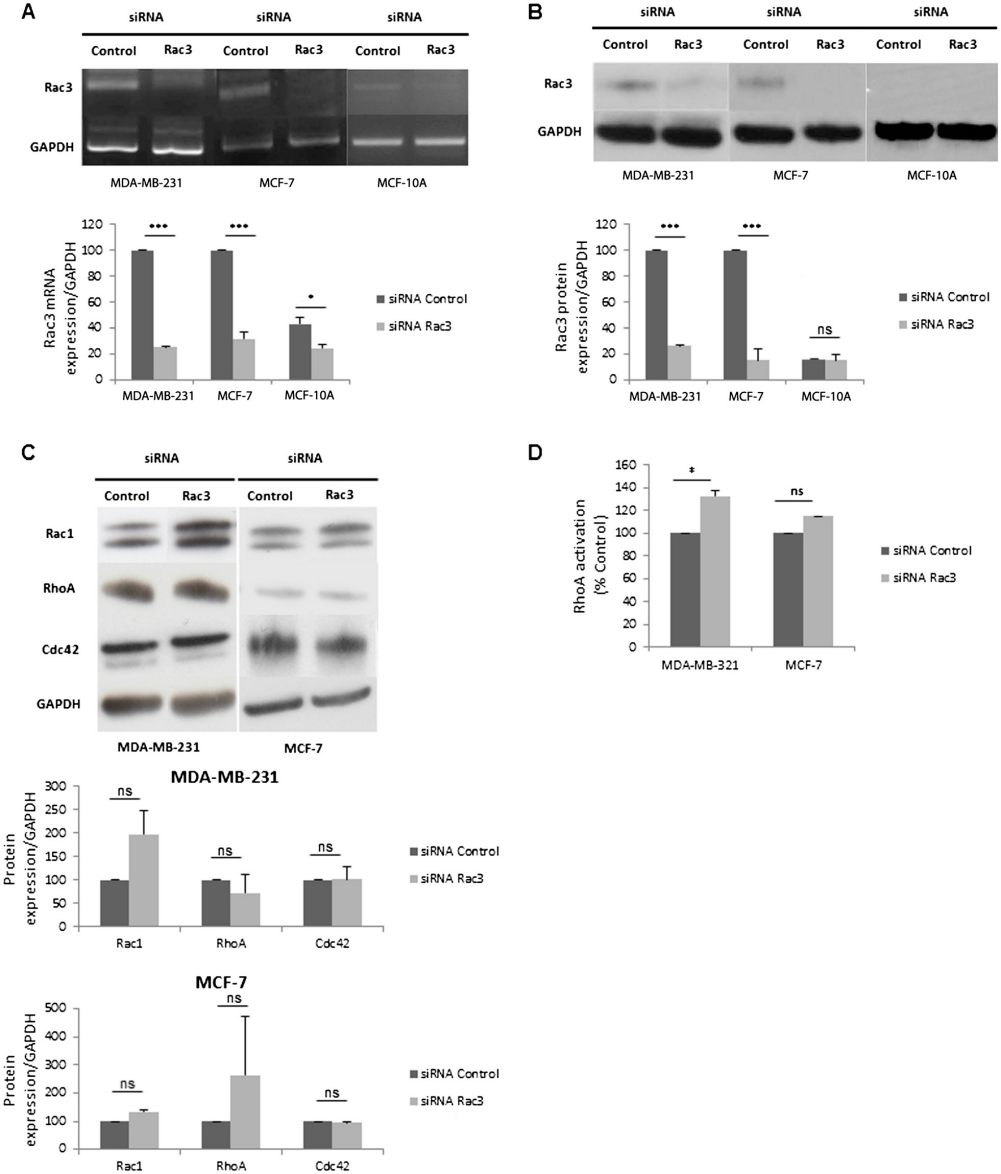
**Efficacy and specificity of siRNA anti-Rac3 treatment and effect on RhoA activation in cancer cells and normal mammary epithelial cells. **Cells were incubated with 10 nM siRNA (control or anti-Rac3) for 48 h or 72 h, proteins or mRNA were then extracted. (**A**) RT-PCR was performed to verify the efficiency of Rac3 siRNA. (**B**) Western blot. (**C**) Western blot performed to verify the specificity of the Rac3 siRNA. (**D**) RhoA activation monitored by G-Lisa. Mean ± S.E., N=3 independent experiments. * P<0.05; ***P<0.001.

We evaluated the expression levels of Rac3 in three cell lines by RT-PCR and western blot. The results showed clear expression of Rac3 mRNA and Rac3 protein in MDA-MB-231 and MCF-7 cell lines. The MCF-10A cells showed only trace amounts of mRNA and no detectable Rac3 protein in western blot (Figure 1A and 1B).

The efficacy of siRNA-mediated inhibition of Rac3 synthesis in these cells was also evaluated (Figure 1A and 1B). In both cancer cell lines, Rac3 siRNA caused a reduction of 75% in Rac3 mRNA and protein levels relative to that seen in cells transfected with a non-targeting control siRNA. This inhibition is specific for Rac3 protein, because anti-Rac3 siRNA did not decrease the expression of Rac1 protein (Figure 1C) despite having 92% homology in amino acid sequence. Expression levels of RhoA and Cdc42 were not modified by anti-Rac3 siRNA treatment (Figure 1C). We tested activated RhoA (Figure 1D) and Cdc42 (data not shown) using a G-Lisa kit. For Cdc42, no significant difference was observed between cells treated with anti-Rac3 and control siRNA in either MDA-MB-231 or MCF-7 (% of control). However, the level of activation of Cdc42 was low in these two cell lines. A slight increase in RhoA activation was found in MDA-MB-231 cells (Figure 1D).

### Effects of Rac3 knockdown on breast cancer cell line morphology, migration, adhesion and invasion

**Figure 2:**
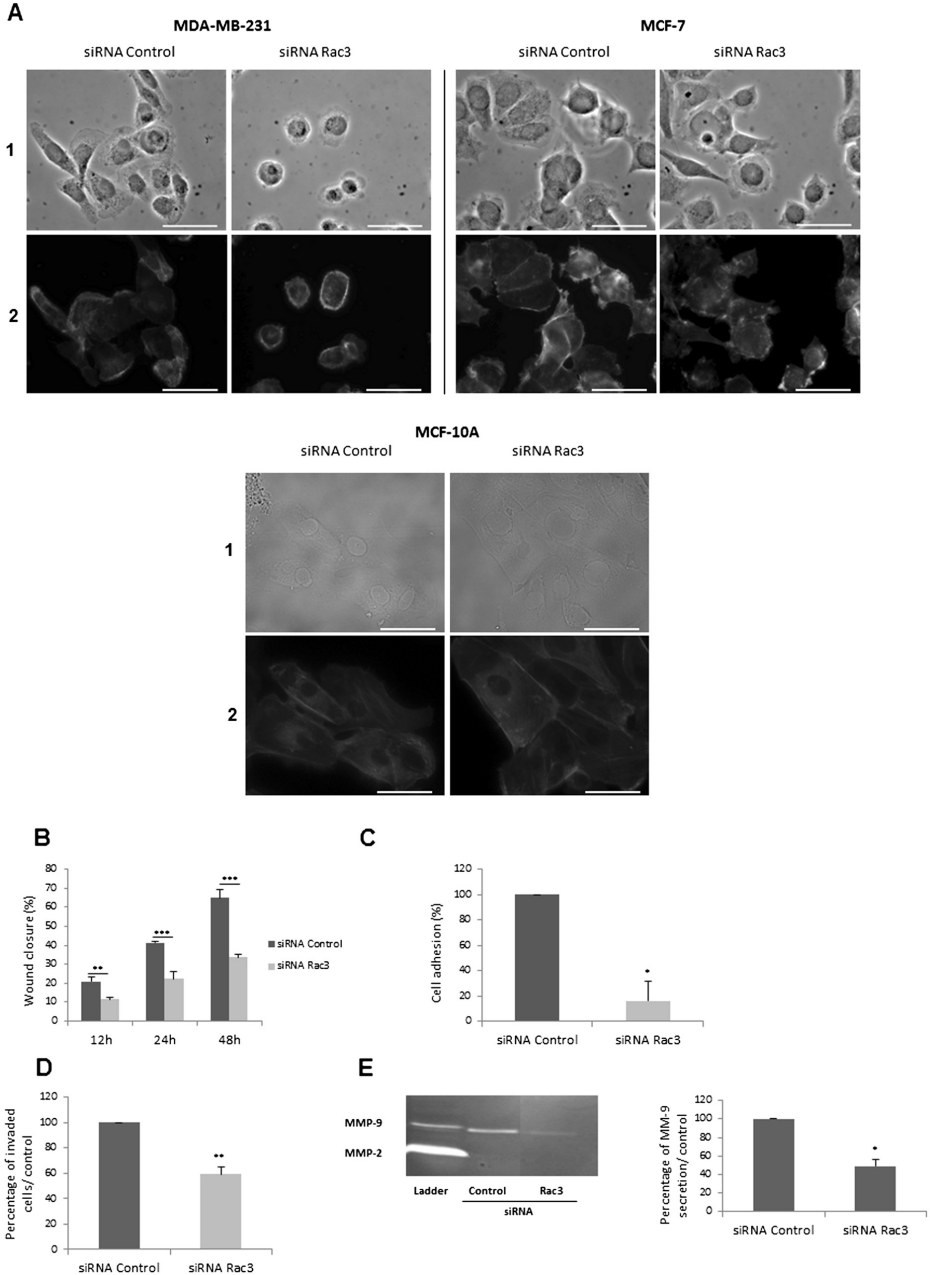
**Effect of Rac3 inhibition on cell morphology, migration, adhesion and invasion. **MDA-MB-231, MCF-7 and MCF-10A cells were treated during 48 h with 10 nM siRNA (control or Rac3). (**A**) Cells were fixed and (A1) cell morphology was observed; (A2) and labelled with phalloidin-TRITC to visualize the actin cytoskeleton (scale bar represents 50 μm). (**B**) Scratch test: wound closure was observed by microcinematography for 48 h; extent of closure at indicated times is expressed as % of initial scratch width. (**C**) Adhesion of MDA-MB-231 to collagen type I matrix under flow conditions. Attached cells were counted and their numbers expressed as % of control. (**D**) Invaded MDA-MB-231 in Boyden chambers were counted and expressed as % of control. (**E**) 48 h conditioned media from siRNA-treated cells (center and right lanes; ladder, left lane) was collected and MMPs present in this medium were detected by zymography; quantification on the right. Mean ± S.E., N=3 independent experiments. *P<0.05; ** P<0.01; ***P<0.001.

As shown in Figure 2A, the MDA-MB-231 cells treated with control siRNA showed spreading, with cell extensions that are involved in adhesion to the extracellular matrix. In contrast, MDA-MB-231 cells treated with Rac3 siRNA became round, no extensions existed and focal adhesions could be observed all around the cells, whereas, in control cells, adhesions were localised at the extremities of the cells. This rounded morphology and the distribution of focal adhesions observed in cells depleted of Rac3 is also valid for MCF-7 cells, but it was less striking, as many cells became round but some of them remained elongated. Depletion of Rac3 in MCF-10A did not affect cell morphology nor actin network organization.

Next, we examined the effect of Rac3 depletion on MDA-MB-231 cell migration, adhesion and invasion. This was not done with the MCF-7 cells because they do not migrate or invade. We observed wound closure in a scratch test (Figure 2B) and found that wound repair proceeded more slowly when cells were treated with anti-Rac3 siRNA than when they were treated with control siRNA. This difference was significant (p<0.01 at 12 h and p<0.001 thereafter).

Moreover, as the attachment of cells to an extracellular matrix (ECM) through integrin receptors modifies gene expression [20], leading to a signaling cascade involved in invasion and metastatic processes, and because integrin clustering is dependent upon small GTPases of the Rho family, we analyzed the consequences of Rac3 silencing on MDA-MB-231 cancer cell adhesion to collagen type I. Adhesion to collagen was tested under flow conditions and in the presence of blood, since hydrodynamic shear forces appear to influence the adhesion of cancer cells to the extracellular matrix. We examined adhesion to collagen type I, since genes involved in adhesion to collagen are consistently up-regulated in metastatic brain tumours in comparison to non-invasive tumours [21]. As seen in Figure 2C, siRNA-mediated down-regulation of Rac3 significantly inhibited MDA-MB-231 cell adhesion to collagen under flow conditions.

For the formation of metastases, both cell adhesion and tissue invasion are needed, so we also tested the effect of Rac3 knockdown on cell invasion in Boyden Chambers coated with Matrigel. To get to the other compartment, cells must first degrade the Matrigel and then change morphology to go through the pores. As shown in Figure 2D, anti-Rac3 siRNA decreased cell invasiveness (40%).

This reduction in cell invasion by Rac3 depletion was associated with a decrease in MMP-9 secretion (Figure 2E), suggesting that inhibition of Rac3 induced a decrease in matrix degradation.

### Effect of Rac3 depletion on vasculogenic mimicry: formation of capillary-like structures on Matrigel by MDA-MB-231 and MCF-7 cells

**Figure 3:**
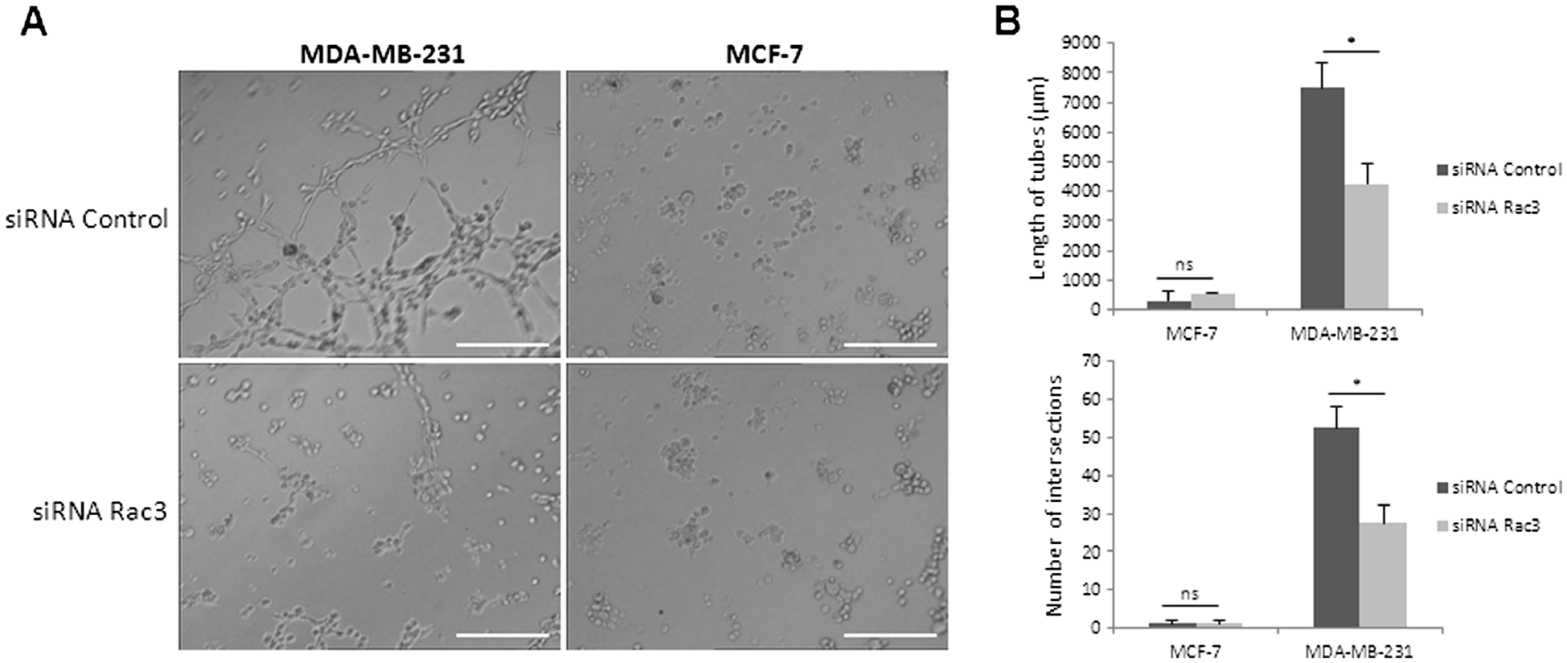
**Effect of Rac3 inhibition on vasculogenic mimicry on growth-factor-rich Matrigel. **Cells were treated for 48 h with 10 nM siRNA (control or Rac3). (**A**) Cells were plated on Matrigel rich in growth factors and photographed 4 h after seeding; scale bar represents 100 μm. (**B**) Cumulative length (top) and intersections (bottom) of capillary tubes were counted. Mean ± S.E., N=3 independent experiments. *P<0.05.

As the vasculogenic mimicry process is a marker of cancer cell aggressiveness, and as it has been shown to depend on MMP-9 secretion [22], the effect of Rac3 depletion on vasculogenic mimicry was also tested. Only MDA-MB-231 cells formed vasculogenic networks when plated on Matrigel (Figure 3A). The capillary-like tubes were observed 4 h after the breast cancer cells were placed on the Matrigel. In contrast, MCF-7 cells were unable to form any capillary-like structures when plated on Matrigel, and VM never occurred even when the incubation time was prolonged up to 18 h: the cell shape remained round (data not shown).

The inhibition of Rac3 in MDA-MB-231 decreased the vasculogenic mimicry. We observed a decrease in both the cumulative length of tubes and the number of intersections (Figure 3B).

### Effects of anti-Rac3 siRNA on MDA-MB-231, MCF-7 and MCF-10A proliferation, survival, and TNFα-induced apoptosis

**Figure 4:**
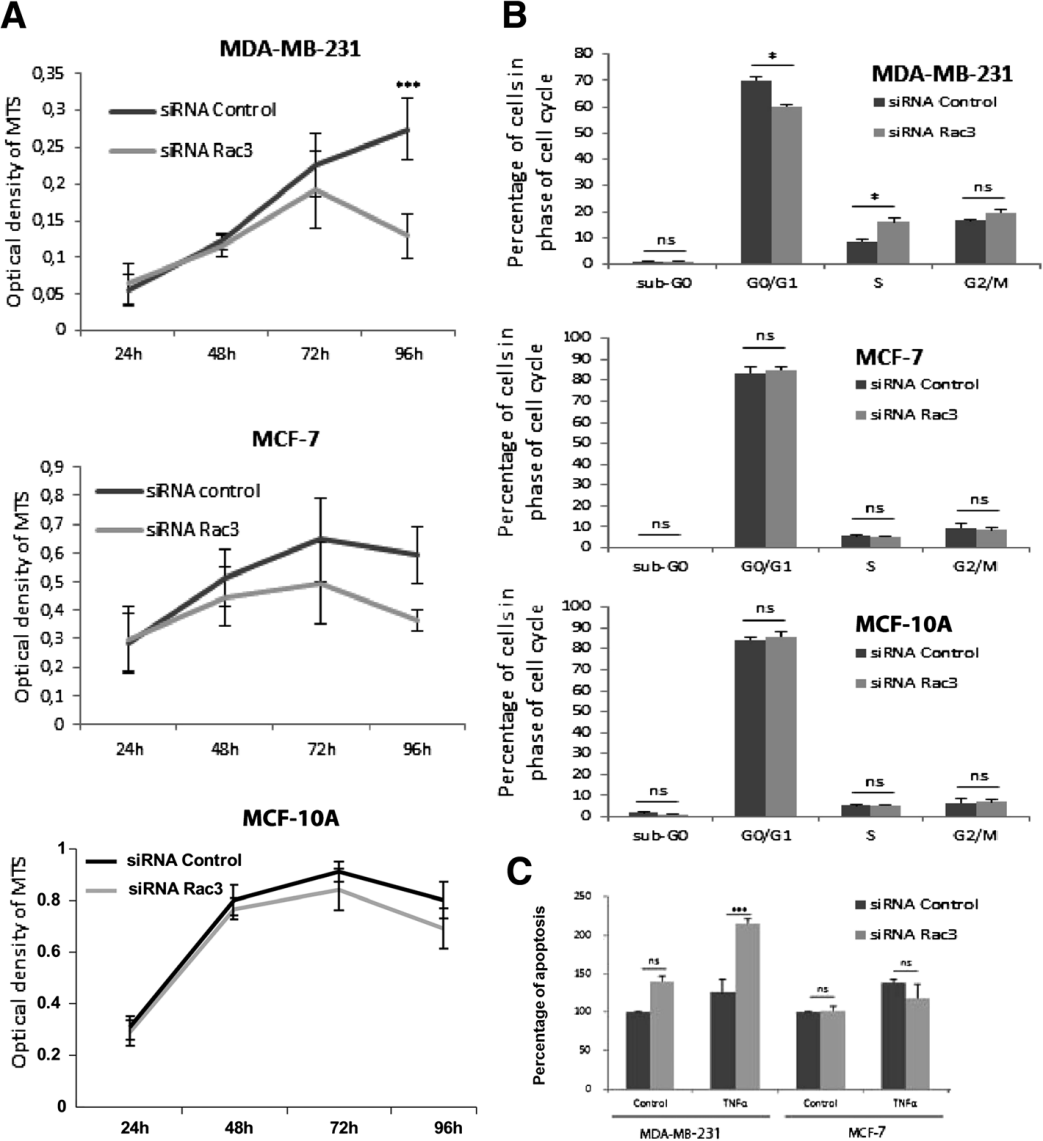
**Effect of Rac3 inhibition on cell proliferation and apoptosis. **MDA-MB-231, MCF-7 and MCF-10A cells were treated with 10 nM siRNA (control or Rac3). (**A**) Cell survival was detected by a MTS assay after 24, 48, 72 and 96 h of treatment. Mean ± S.E., N=5 independent experiments. *** P<0.001 (**B**) After 96 h siRNA treatment, distribution of cells in the cell cycle was quantified by flow cytometry. Mean ± S.E., N=2 independent experiments. *P<0.05 (**C**) After 48 h siRNA treatment, apoptosis was first induced by TNFα (50 ng/ml) during an additional 24 h, then quantified by ELISA detection of histone/DNA complex in the cytoplasm. Mean ± S.E., N=3 independent experiments. ***P<0.001 relative to the control siRNA value, defined as 100%.

Transfection of breast cancer cell lines with siRNA anti-Rac3 significantly decreased the proliferation of MDA-MB-231 but not MCF-7 cells after 72 h and 96 h of treatment. Additionally, siRNA anti-Rac3 did not significantly affect MCF-10A cells, probably due to their very low expression of Rac3 (Figure 4A).

In addition, the inhibition of Rac3 in MDA-MB-231 induced a cell cycle arrest in S phase after 96 h of treatment. In contrast, no cell cycle arrest was detected in MCF-7 or MCF-10A (Figure 4B).

As shown in Figure 4C, treatment of MDA-MB-231 with anti-Rac3 siRNA for 48 h, prior to a 24 h incubation with TNFα to induce apoptosis, provoked a significant increase in apoptosis: the apoptotic index was 210% of that seen in cells treated with control siRNA. In contrast, no significant change was observed in MCF-7 cells under the same conditions.

### Effects of anti-Rac3 siRNA on MDA-MB-231 and MCF-7 cell signaling

**Figure 5:**
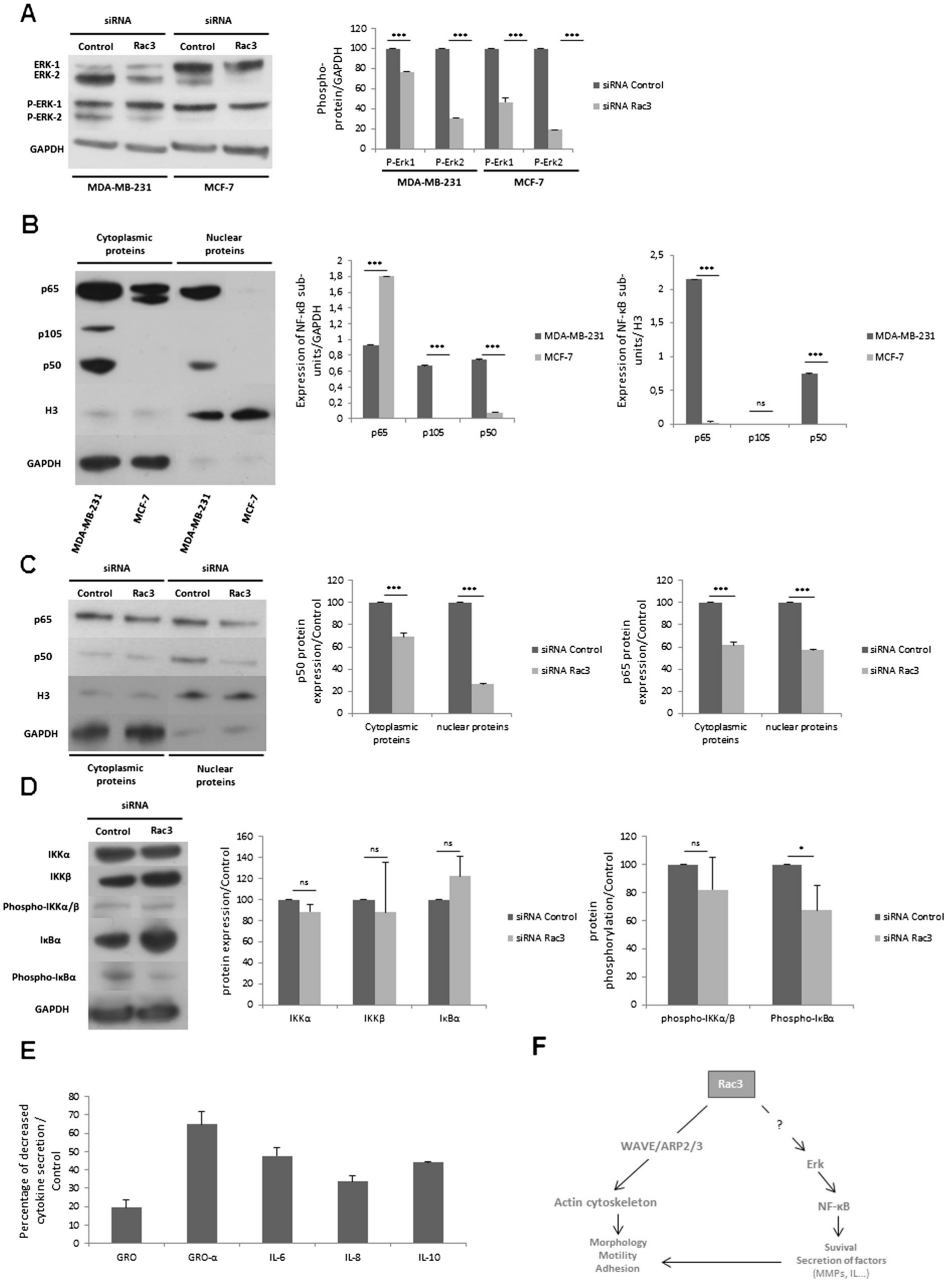
**Effect of Rac3 depletion on cell signaling. **Cells were treated with 10 nM siRNA (control or Rac3). After 48 h of treatment, (**A**) total proteins were extracted and ERK-1, ERK-2 and their respective phosphorylated forms detected by western blot. (**B**) Cytoplasmic and nuclear proteins were extracted, and expression of NF-κB subunits detected by western blot. GAPDH is a control for cytoplasmic protein extract and H3 is a control for nuclear extract (**C**) Cytoplasmic and nuclear proteins were extracted from MDA-MB-231, and NF-κB p65 and p50 subunits detected by western blot. GAPDH is a control for cytoplasmic protein extract and histone H3 is a control for nuclear extract. (**D**) Total proteins were extracted from MDA-MB-231 and proteins of NF-κB activation pathways were detected by western blot. (**E**) Action of siRNA anti-Rac3 on cytokine secretion by MDA-MB-231 was detected by cytokine array. Mean ± S.E. N=3 independent experiments. * p<0.05; *** P<0.001.

To understand the biological mechanisms responsible for the effects of Rac3 on cell aggressiveness, we examined the effect of Rac3 inhibition on ERK and NF-κB activation (Figures 5A and 5B, respectively). ERK-1 and ERK-2 targets were selected because, in a preliminary study with a cell array for examining cell signaling pathways, only the phosphorylation of ERK-1 and −2 was inhibited in cells treated with anti Rac3 siRNA (data not shown).

NF-κB was also analyzed, to understand the effects of Rac3 inhibition on the increase of apoptosis induced by TNFα. Indeed, it has been established that TNFα is responsible both for apoptosis, when the NF-κB pathway is not activated, and for cell survival, when NF-κB is activated. These differences in cell behavior with respect to TNFα are dependent upon the nature of the adaptor molecules recruited inside the cells.

In this study, we observed that the depletion of Rac3 in both MDA-MB-231 and MCF-7 cell lines resulted in a decreased ERK activation and total ERK protein levels. In MDA-MB-231, the decrease of activation concerned essentially ERK-2 protein (70% inhibition compared to the control). In MCF-7, the activation of both ERK-1 and ERK-2 was decreased (53% for ERK-1 and 81% for ERK-2) (Figure 5A). However, we found (Figure 5B) that p65 and p50 subunits of NF-κB are active only in MDA-MB-231, not in MCF-7. The presence of p105 subunits only in the cytoplasmic fraction indicates that the nuclear fraction is clean. Figure 5C shows that in MDA-MB-231 the activation of both the p65 and p50 subunits of NF-κB was inhibited by Rac3 siRNA treatment, as shown by the reduction of levels of both subunits in the nucleus.

Figure 5D shows that Rac3 inhibition in MDA-MB-231 induces a decrease in IκBα phosphorylation.

To complete this investigation of the molecular mechanism of Rac3 action in cancer cell aggressivity, we also analyzed the profile of cytokine secretion by MDA-MB-231 cells (Figure 5E). We observed that the depletion of Rac3 induced decreased secretion of GRO (19% compared to the control), GROα (65%), IL-6 (47%), IL-8 (34%) and IL-10 (44%).

## Discussion

Rho- and Rac-GTPases are known to be implicated in cancer cell aggressivity. We established previously that RhoA is over-expressed and spontaneously activated in MDA-MB-231, and found that blockage of RhoA expression inhibited cancer cell invasion and tumour growth by more than 80% in a mouse model [23]. The role of Rac1 in cancer progression has also been clearly shown [4]. In contrast, the role of Rac3 in the aggressiveness of breast cancer cells is not well established: in some studies Rac3 was found to be involved mainly in cell proliferation [24], whereas in others Rac3 has been implicated in cell invasion but not migration [25]. Despite 92% amino acid homology between Rac1 and Rac3 proteins, they differ in their C-terminal membrane targeting regions, which are known effector-binding regions, suggesting that differential effector binding could occur for Rac1 versus Rac3 in different cell types [8]. Moreover, in neuronal cells [26], Rac1 and Rac3 are known to have opposing functions in cell morphology and adhesion to the ECM.

Our aim in this study was to examine the effects of Rac3 in breast cancer cell aggressivity in both the invasive MDA-MB-231, which have a spontaneous activation of RhoA, and in the non-invasive MCF-7, which do not. Furthermore, we determined that even after stimulation, the activated Rho found in MCF-7 represented 48% of that seen in MDA-MB-231 (data not shown). The criteria we defined as factors of aggressiveness were proliferation, migration, invasion, adhesion on extracellular matrix in flow conditions, and vasculogenic mimicry. To examine the role of Rac3, we analyzed the consequences of Rac3 depletion by using siRNA technology.

Firstly, to validate our work, we studied the efficacy and specificity of Rac3 siRNA. Under the conditions used, the siRNA treatment is indeed efficient. This effect is specific, because anti-Rac3 siRNA did not inhibit the expression of Rac1, RhoA or Cdc42 (Figure 1C). Moreover, the possible interrelations between the different Rho and Rac proteins led us to examine the effects of Rac3 inhibition on activation of other Rho proteins that could potentially influence the aggressiveness cancer cells. In both cell lines, activated Cdc42 was not modified by Rac3 depletion, whereas RhoA activation was slightly increased. This increase did not seem to be enough to counterbalance the Rac3-depletion-induced cell inhibition effects. Therefore, the effects of Rac3 depletion on MDA-MB-231 and MCF-7 cells cannot be attributed to a modification of Rho or Cdc42 expression or activity.

Secondly, we found that Rac3 depletion in MDA-MB-231 cells inhibited cell spreading and lamellipodia extension (Figure 2A), leading to cell rounding associated with a disorganisation of the actin cytoskeleton. The rounding of MDA-MB-231 is consistent with a loss of lamellipodia, which are involved in cell adhesion to the ECM by connecting the ECM to actin filaments. Our results are in agreement with the results of Joyce and Cox [27], who reported that both Rac1 and Rac3 strongly stimulated lamellipodia formation in fibroblasts. However, the effect is much clearer in MDA-MB-231 than in MCF-7 cells. As the actin network is more organized in MDA-MB-231 than in MCF-7, this can explain the differences of impact of Rac3 inhibition on cell morphology because Rac3 regulates actin cytoskeleton organization (Figure 5F). At any rate, this disappearance of lamellipodia and cell rounding in MDA-MB-231 when Rac3 is depleted provides an explanation for the important decrease in their adhesion to collagen type I under flow conditions. This cell adhesion has been implicated in the development and progression of the majority of cancers [28]. Moreover, the observed decrease in adhesion might be expected to prevent metastatic dissemination to bone, since collagen type I is the most abundant protein in the bone matrix, and cell adhesion on collagen is considered to be a marker of tumour invasiveness in bone [29].

In addition, the depletion of Rac3 induced an important decrease in MDA-MB-231 migration and invasion through Matrigel. The cell rounding we observed upon Rac3 siRNA treatment may be partially responsible for the decreased cell invasion (Figure 2D) and speed of wound repair in the scratch test (Figure 2B), since it has been reported that small pseudopodia are required for cell motility and to bear focal complexes [30,31]. The decrease in MMP-9 secretion observed in Rac3-depleted cells can also contribute to the reduction of cell invasion through Matrigel.

Moreover, we tested the effect of Rac3 depletion on capillary-like structure formation (VM) by cancer cells plated on Matrigel rich in growth factors. This cell property is currently considered as being a sign of great aggressiveness, possibly by contributing to the blood supply in the tumours [32]. This is confirmed by our observation showing that, whereas the aggressive cells (MDA-MB-231) were able to form capillary-like tubes in Matrigel, the poorly aggressive cells, MCF-7, did not. To form channels, cancer cells must migrate and modify their morphology to become elongated. The inhibition of Rac3 expression in MDA-MB-231 cells blocked the VM; this provides yet another argument for thinking that Rac3 plays a role in tumour aggressiveness, even in cells where the small GTPase RhoA is overexpressed and spontaneously activated. The decrease in MMP-9 secretion following treatment with siRNA anti-Rac3 can also be involved in its inhibitory effect on VM in MDA-MB-231 cells, as it was described that both MMP-2 and MMP-9 play an important role in VM in cancers [33].

We also analyzed the effect of Rac3 inhibition on two other functions important in cell aggressivity: cell proliferation and resistance to apoptosis. siRNA anti-Rac3 induced a slight decrease in cell proliferation in MDA-MB-231 cells between 72 and 96 hours after treatment. To understand this decrease of cell number we first analyzed the effects of Rac3 inhibition on cell apoptosis. We did not observe any modification of the apoptotic index under basal conditions for either MDA-MB-231 or MCF-7 cell lines. However, in MDA-MB-231 the Rac3 inhibition increased sensitivity to TNFα-induced apoptosis. This effect on apoptosis does not occur in MCF-7. Moreover, when we monitored the progression of treated cells through the cell cycle, we found that MDA-MB-231cells were blocked in S phase but no modification was observed for MCF-7. This can explain the more profound effect of Rac3 inhibition on proliferation in MDA-MB-231compared to MCF-7.

All these results intrigued us because, although MCF-7 cells express Rac3, they are poorly aggressive and non-invasive compared to MDA-MB-231. Consequently, to understand why Rac3 plays a positive role in MDA-MB-231 aggressiveness but does not do so in MCF-7 cells, we analyzed the effects of Rac3 depletion on cell signaling. We found that, in both cell types, Rac3 inhibition led to a decrease of ERK and phospho-ERK protein levels. The decrease of the level of ERK protein in the cell could be due to an inhibition of ERK expression or ERK stabilisation. We therefore analyzed the consequences of ERK inactivation for MDA-MB-231 and MCF-7 cells.

Knowing that ERK is critical for the activation of NF-κB, we postulated the intervention of a cell signal cascade, Rac3/ERK/NF-κB, analogous to that previously proposed for Rac1 [34]. Indeed, activated ERK-1 and ERK-2 are known to act upstream of IκB kinase activation, initiating the degradation of IκB through the ubiquitin/proteasome pathway, leading to NF-κB activation [35].

In MDA-MB-231 cells, we found that Rac3 does indeed induce cell signaling leading to NF-κB activation by an increase in IκBα phosphorylation and degradation. This NF-κB activation is important both for protection against apoptosis and for MMP-9 secretion [36], which is implicated in cancer cell invasion [37] and vasculogenic mimicry, two major criteria of cancer cell aggressiveness; and activated NF- κB represses apoptosis by inducing the expression of anti-apoptotic genes including cIAPs, FLIP, TRAF-1, TRAF-2, Bcl-2, and Bcl-xL [38]. In contrast, in MCF-7, Rac3 does not increase cancer aggressivity despite the fact that Rac3 activates ERK in these cells. This could be explained by the fact that NF-κB p50 and p65 proteins are both inactive in MCF-7 (Figure 5B). Thus although Rac3 is expressed and does activate ERK in MCF-7, this does not result in increased NF-κB activation, because of the very small amounts of the two active subunits of NF-κB expressed in these cells. This also helps explain why the MCF-7 expressing Rac3 are not very aggressive, and show relatively weak invasion, migration and VM.

Finally, we analyzed the importance of ERK/NF-κB signaling in the Rac3-depletion-induced modification of cytokine secretion in MDA-MB-231 cells. We found that IL-6, IL-8, GRO, GRO-α and IL-10 were all down-regulated in Rac3-depleted cells. These cytokines are known to be secreted by immune cells; however, it has been reported that they can also be secreted by cancer cells, and their secretion is usually associated with a poor prognosis [39-41].

Interestingly, IL-6, IL-8 and GRO, are involved in tumour aggressivity by favoring a higher invasiveness potential of cancer cells and a proangiogenic activity [42,43]. The decrease of the secretion of these three cytokines by Rac3-depleted MDA-MB-231 could be explained by the role of Rac3 in ERK-2/NF-κB signaling. In fact, the levels of IL-6, IL-8 and GROα are under the control of NF-κB [37,44,45]. Moreover, a recent work has underlined the essential role of IL-8 signaling for the acquisition and/or maintenance of the mesenchymal and invasive features of overexpressing tumour cells and shows that IL-8 secreted by tumour cells undergoing epithelial-mesenchymal transition can potentiate tumour progression [46]. The decrease in IL-10 secretion induced by anti-Rac3 siRNA can also be explained by the action of Rac3 on ERK-2/NF-κB signaling, but its role in cancer aggressivity has been reported to be due mainly to an immunosuppression and also to its angiogenic activity [47].

We also studied the effect of Rac3 knockdown in breast epithelial cells and showed in these cells that Rac3 inhibition had not effect on the cell proliferation, morphology, actin organization and cell cycle evolution. These results were probably due to the low level of Rac3 protein expressed in these cells. Mira *et al.*[24] has previously demonstrated the crucial role of Rac3 in breast cell proliferation by introduction of hyperactive Rac3 into normal breast epithelial cell line and breast cancer cell lines. They showed that hyperactive Rac3 contributes to the aggressive growth of epithelial cancer cells [24]. Therefore, it appears that the non-aggressive behaviour of normal epithelial cells can be related to absence of hyperactivity or low expression of Rac3. Inversely, aberrant Rac3 expression and activation, along with the effectiveness of its downstream pathways in cancer cells, contribute to cancer aggressiveness.

## Conclusions

There have been few studies on the effects of Rac3 on the aggressiveness of breast cancer cells, and the results obtained do not always agree. In this study, we investigated the effects of Rac3 on the aggressiveness of two breast cancer cell lines that show similar levels of Rac3 expression: 1) MDA-MB-231, which is highly aggressive, and for which we had previously established that the increased expression and spontaneous activation of RhoA plays a role in the aggressiveness, and 2) MCF-7, a poorly aggressive and non-invasive cell line. We show here that, even in MDA-MB-231 cells, where RhoA is spontaneously activated, Rac3 plays a significant role in cell proliferation, adhesion to collagen, migration, and invasion, and ultimately decreases the cellular apoptosis response to TNFα. This last effect is mediated by Rac3/ERK2/NF-κB signaling. In contrast, Rac3 has minimal effect in MCF-7 cells under the same conditions, despite activating ERK. This can be explained by the fact that the signaling pathway cannot be fully effective because of the inactive state of NF-κB proteins in these cells. To induce MDA-MB-231 aggressiveness, Rac3 seems to be implicated in at least two signaling pathways. It can regulate actin cytoskeleton organization by activating WAVE/Arp2/3 proteins, and then induce lamellipodia formation, which is necessary for cell dissemination, as described for Rac subgroup proteins [48]. This cytoskeleton regulation by Rac3 is not sufficient for cell dissemination, but activation of ERK/NF-κB signaling by Rac3 leads to an increase of cell survival, proliferation and secretion of cytokines implicated in pro-invasive and/or pro-angiogenic activity. Interestingly, in the non tumorigenic cell line MCF-10A, Rac3 seems not to play a critical role. This can be due to the low level of Rac3 expression level of Rac3 in these cells.

Our results thus confirm the crucial role of Rac3 in breast cancer aggressiveness and show the potential usefulness of Rac3 depletion in breast cancer therapy.

## Abbreviations

BSA: Bovine Serum Albumin; ECM: Extracellular Matrix; ER: Estrogen Receptor; FBS: Foetal Bovine Serum; MMP: Matrix MetalloProteinase; NF-κB: nuclear factor kappa-light-chain-enhancer of activated B cells; TNF: Tumor Necrosis Factor; VM: Vasculogenic Mimicry.

## Competing interest

The authors declare that they have no competing interest.

## Authors’ contribution

CG was responsible for this study, designing and executing experiments, interpreting the results and contributing to drafting the manuscript. UJ participated in the analysis and interpretation of the results, was involved in writing the manuscript and provided assistance to CG for the experiments. LH participated in development, analysis and interpretation of experiments in MCF-10A. LLP contributed to analysis and interpretation of the results and was extensively involved in writing the manuscript. AP participated in design, development, analysis and interpretation of cell cycle experiments and provided assistance for western blot of NF-κB pathway. CB participated in development and executing of western blot of NF-κB pathway and provided assistance for micro-cinematography experiment. CQH, CR and ML provided assistance to CG for the experiments. FFL participated in design, development, analysis and interpretation of cell adhesion experiment. LC and JPV provided general intellectual support and participated in data discussions. HLu participated in the design of this study, in analysis and interpretation of the results, and in development of the study methods. JS and RV participated in study development and critically reviewed the data, contributing to interpretation of the results and writing the manuscript. CS initiated and supervised this study, participating in its design and development, analyzing and interpreting the results, drafting the manuscript and coordinating and overseeing all but the last of revision of the manuscript. HLi was responsible for this study, participating in its design and development, analyzing and interpreting the results, drafting the manuscript and coordinating and overseeing all stages of revision of the manuscript. All authors read and approved the manuscript.

## Funding

This work was supported by the Groupement des Entreprises Francaises dans la Lutte contre le Cancer de Rouen (GEFLUC of Rouen); and the Ligue Regionale de Haute Normandie de Lutte contre le Cancer.

Caroline Gest is a recipient of grant from Ministère de l’enseignement supérieur et de la recherche. We are grateful to the Ligue Régionale de l’Eure contre le Cancer; and to the Association Ti’toine, for financing the fellowship of Ulrich Joimel.

## Pre-publication history

The pre-publication history for this paper can be accessed here:

http://www.biomedcentral.com/1471-2407/13/63/prepub
